# B Cells Enhance Antigen-Specific CD4 T Cell Priming and Prevent Bacteria Dissemination following *Chlamydia muridarum* Genital Tract Infection

**DOI:** 10.1371/journal.ppat.1003707

**Published:** 2013-10-31

**Authors:** Lin-Xi Li, Stephen J. McSorley

**Affiliations:** Center for Comparative Medicine, Department of Anatomy, Physiology and Cell Biology, School of Veterinary Medicine, University of California, Davis, Davis, California, United States of America; University of Pennsylvania, United States of America

## Abstract

B cells can contribute to acquired immunity against intracellular bacteria, but do not usually participate in primary clearance. Here, we examined the endogenous CD4 T cell response to genital infection with *Chlamydia muridarum* using MHC class-II tetramers. *Chlamydia*-specific CD4 T cells expanded rapidly and persisted as a stable memory pool for several months after infection. While most lymph node *Chlamydia*-specific CD4 T cells expressed T-bet, a small percentage co-expressed Foxp3, and RORγt-expressing T cells were enriched within the reproductive tract. Local *Chlamydia*-specific CD4 T cell priming was markedly reduced in mice lacking B cells, and bacteria were able to disseminate to the peritoneal cavity, initiating a cellular infiltrate and ascites. However, bacterial dissemination also coincided with elevated systemic *Chlamydia*-specific CD4 T cell responses and resolution of primary infection. Together, these data reveal heterogeneity in pathogen-specific CD4 T cell responses within the genital tract and an unexpected requirement for B cells in regulating local T cell activation and bacterial dissemination during genital infection.

## Author Summary

Sexually transmitted infections caused by *Chlamydia* are increasing every year in the US and an effective vaccine is urgently required. Unfortunately, we currently only have a rudimentary understanding of the natural host immune response to *Chlamydia* infection, especially in the context of the female genital tract. Here, we have developed new reagents that allow direct visualization of the host T cell responses to vaginal *Chlamydia* infection using a mouse model of infection. These new tools reveal an unexpectedly complex CD4 T cell response to infection and a surprising role for B cells in preventing the spread of bacteria to multiple host tissues. This greater understanding of the host response to infection may eventually allow the construction of an effective vaccine.

## Introduction

*Chlamydia trachomatis* is an obligate intracellular pathogen that causes the most prevalent bacterial sexual transmitted infection worldwide [1]. In the US, *Chlamydia* is now the most common notifiable disease reported to the US Centers for Disease Control (CDC). The 1.4 million cases of *Chlamydia* infection reported in 2011 represent an 8% increase over the previous year and is the largest number of annual infections ever reported to the CDC for any condition [2]. The introduction of a *Chlamydia* screening and control program in the mid-1990s has not prevented annual increases in infection, although a portion of this increase is due to improved disease surveillance [3]. Overall, the CDC reports a median 8.3% *Chlamydia* positivity test among women aged 15–24, making this one of the most prevalent bacterial infections in the US.

Most *Chlamydia* infections are initially asymptomatic and therefore unlikely to be treated. However, 5–15% of females with untreated infection will eventually develop serious pelvic inflammatory disease (PID) as a consequence. Furthermore, 1 in 6 women who develop PID will become infertile, and many others will develop chronic pelvic inflammation and pain, or suffer from ectopic pregnancy [4]–[6]. The combination of an extraordinarily high number of infections, the asymptomatic nature of initial disease, and the potential for serious reproductive pathology in young women, means that *Chlamydia* is now recognized as a growing health care problem in the US. The current consensus among scientists and clinicians is that an effective *Chlamydia* vaccine is urgently needed [7].

The development of an effective *Chlamydia* vaccine would likely alleviate the burden of *Chlamydia* on the public health care system. However, the rational design of a *Chlamydia* vaccine would be aided by improved understanding of the cellular immune response to infection of the female reproductive tract. As *Chlamydia* is an obligate intracellular pathogen, IFN-γ production by CD4 Th1 cells is essential for protective immunity to primary and secondary infection [8]–[13]. Unfortunately, we have at present only a rudimentary understanding of the development of protective Th1 responses in the context of the female upper reproductive tract and the extent of T helper heterogeneity is unclear. One of the major roadblocks to improving this situation is the lack of antigen-specific reagents that would allow detailed investigation of *Chlamydia*-specific CD4 T cell responses using a relevant genital challenge model.

Previous studies have demonstrated that antibody production by B cells can assist protective immunity during secondary *Chlamydia* infection [14]–[16]. In contrast, B cells are thought to be dispensable for resolving primary *Chlamydia* infection, and B cell-deficient and wild type mice shed similar numbers of *Chlamydia*, as measured by vaginal swabs [17], [18]. However, another study using the respiratory route of infection demonstrated that intranasal infection with *Chlamydia* requires B cells for efficient CD4 T cell activation [19]. Therefore, the issue of whether B cells contribute to initial CD4 T cell priming during vaginal infection requires additional analysis.

In this study, we generated MHC class-II tetramers to visualize the endogenous CD4 T cell response to systemic and genital tract *Chlamydia* infection. We show that, unlike intravenous infection, reproductive tract infection is associated with a short delay in the clonal expansion of *Chlamydia*-specific CD4 T cells in the local draining lymph node. While almost all expanded CD4 T cells expressed the Th1 marker, T-bet, we detected an expanded pool of *Chlamydia*-specific Tregs that co-expressed T-bet and Foxp3, and a population of *Chlamydia*-specific Th17 cells that were specifically enriched in the reproductive tract. In addition, we noted a surprising requirement for B cells in *Chlamydia*-specific CD4 cell priming within local draining lymph nodes. Loss of local priming in the absence of B cells coincided with bacterial dissemination to the peritoneal cavity inducing inflammatory infiltrate and ascites. Together, these data demonstrate heterogeneity in *Chlamydia*-specific T helper responses and an unexpected role for B cells in local CD4 T cell priming and bacterial containment within the upper reproductive tract.

## Results

### Visualization of *Chlamydia*-specific CD4 T cell expansion after intravenous infection

**Figure 1:**
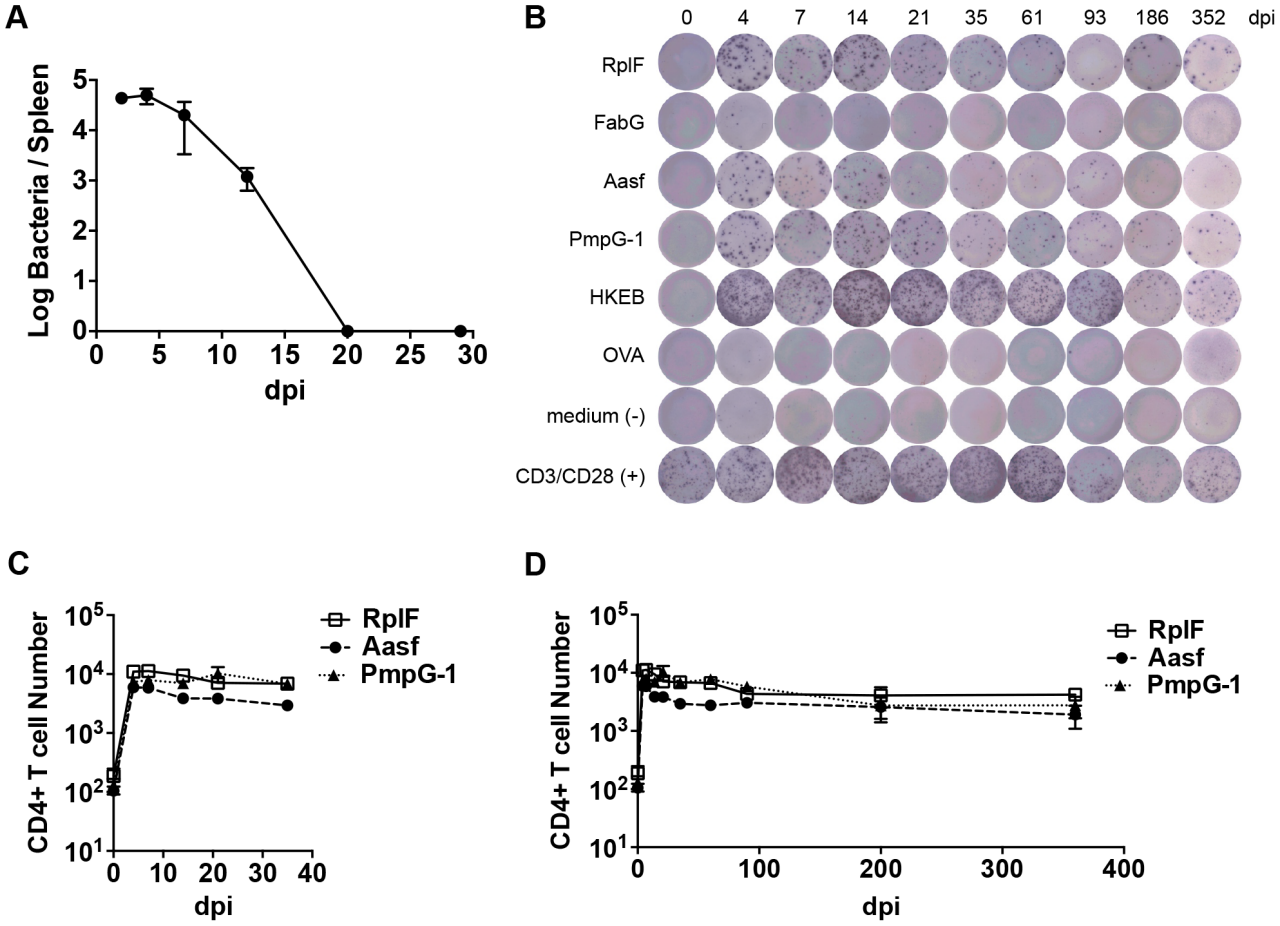
Kinetics of antigen-specific CD4^+^ T cell expansion after *C. muridarum* intravenous (i.v.) infection. C57BL/6 mice were infected intravenously with 1×10^5^ *C. muridarum*. At various time points post infection, spleen IFU was enumerated by plating supernatant from tissue homogenize on HeLa229 cells. (A) CD4 T cells from the spleens and lymph nodes were purified and stimulated with 10 µM of *C. muridarum* peptides or 1×10^5^ HKEBs for 20 h in the presence of irradiated splenocytes. IFNγ production was measured by ELISPOT assay. (B) Representative image of IFNγ ELISPOT plates at each time point measured. (C) and (D) Graphs summarize the total number of IFNγ-producing CD4 T cells per infected mouse. Data shown are pooled results from two independent experiments with eight mice per time point. Error bars show mean number ± SEM.

In order to develop an overview of the adaptive response to *Chlamydia* infection, we initially examined the kinetics of bacterial growth and *Chlamydia*-specific CD4 T cell expansion during systemic infection with *Chlamydia*. When C57BL/6 female mice were infected intravenously (i.v.) with 1×10^5^ inclusion-forming units (IFUs) of *C. muridarum*, the bacterial burden in the spleen peaked around day 4 post-infection and decreased quickly thereafter (Fig. 1A). At day 20, no infectious *Chlamydia* was detected in the spleen (Fig. 1A). Consistent with previous findings [20], a small number of *Chlamydia* were found in the lung during the first week of systemic infection, but no bacteria were detected in kidney or heart at any time point (data not shown).

Numerous *C. muridarum* MHC class-II epitopes have been uncovered by Immunoproteomic analysis of infected APCs [21]. We used an ELISPOT assay to monitor the frequency of CD4 T cells responding to multiple *C. muridarum* epitopes after systemic infection. A population of IFN-γ-secreting CD4 T cells responding to RplF_51–59_, Aasf_24–32_, and PmpG-1_303–311_ was detected as early as 4 days after infection (Fig. 1B and C). Expansion of IFN-γ-secreting CD4 T cells peaked around day 4–7, and was followed by a slow contraction of the population over the next 90 days, before a plateau was reached that lasted for at least 352 days (Fig. 1B and 1D). Thus, peak expansion of IFN-γ-secreting CD4 cells closely mirrored peak bacterial burdens in vivo, and stable *Chlamydia*-specific CD4 T cell memory frequencies were maintained in the absence of active *Chlamydia* infection.

### Construction of *Chlamydia*-specific pMHCII tetramers

**Figure 2:**
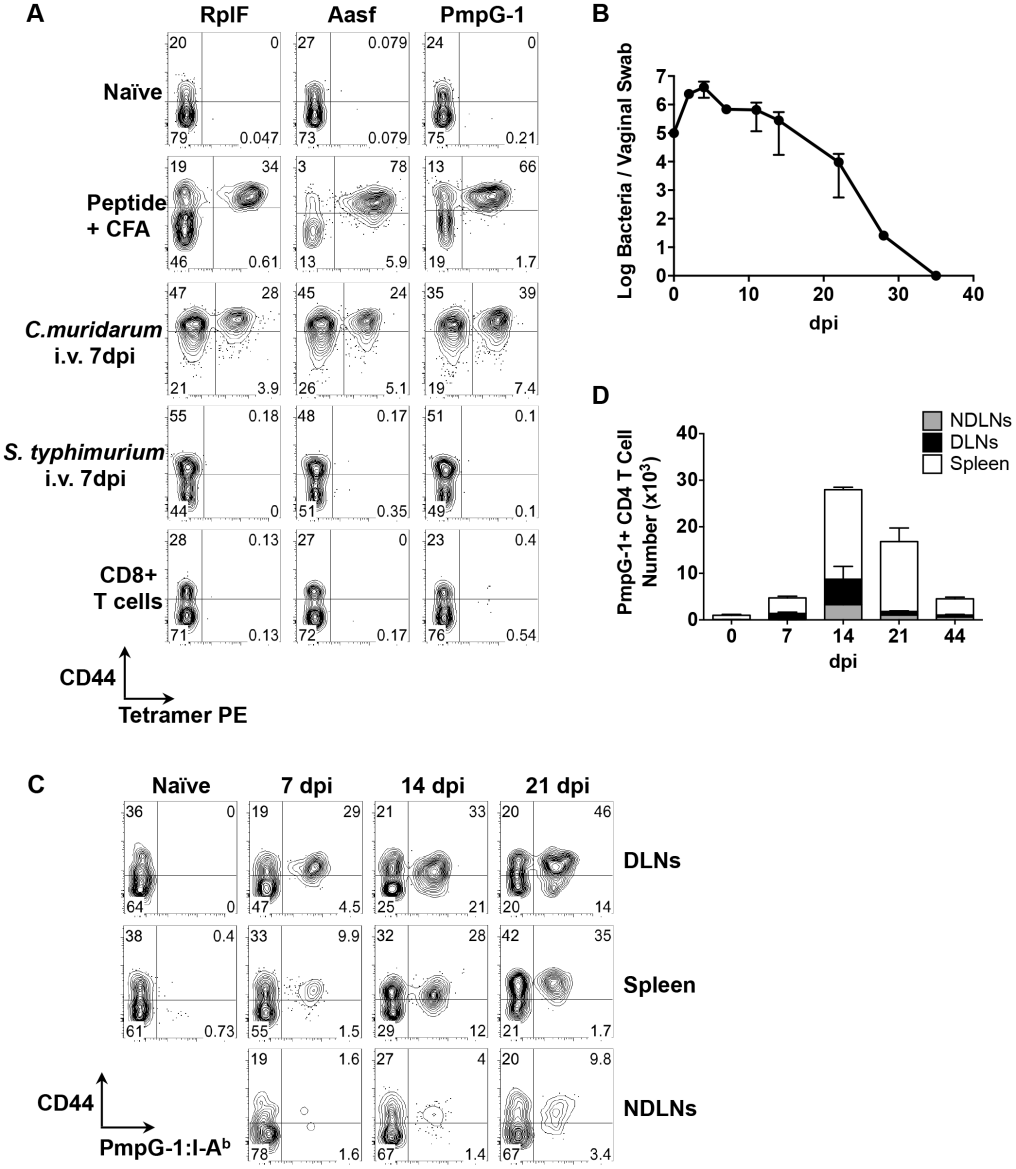
Kinetics of antigen-specific CD4^+^ T cell expansion after *C. muridarum* intravaginal (i.vag.) infection. (A) C57BL/6 mice were immunized subcutaneously with 100 µg of *C. muridarum* peptide (RplF_51–59_, Aasf_24–32_ or PmpG-1_303–311_) in the presence of CFA or infected intravenously with either 1×10^5^ *C. muridarum* or 5×10^5^ *S. typhimurium*. Seven days post immunization/infection, axillary, brachial and inguinal lymph nodes were isolated from peptide-immunized mice, spleen and lymph nodes were harvested from *C. muridarum* or *S. Typhimurium* infected mice and endogenous RplF, Aasf or PmpG-1-specific CD4 T cells were detected with RplF_51–59_:I-A^b^, Aasf_24–32_:I-A^b^ or PmpG-1_303–311_:I-A^b^ tetramers, respectively. FACS plots showing representative results of tetramer staining after fluorescent microbeads enrichment. All plots were pre-gated on CD11b^−^CD11c^−^F4/80^−^B220^−^CD3^+^CD4^+^ cells except CD8^+^ T cells (bottom row), which were gated on CD11b^−^CD11c^−^F4/80^−^B220^−^CD3^+^CD8^+^ cells. (B–D) C57BL/6 mice were infected i.vag. with 1×10^5^ *C. muridarum*. (B) At various time points post infection, bacteria burden at lower genital tract was measured by vaginal swabs. (C) Endogenous PmpG-1-specific CD4 T cells in the spleen, draining lymph nodes and non-draining lymph nodes were detected by PmpG-1_303–311_:I-A^b^ tetramers. FACS plots showing representative results of tetramer staining after magnetic enrichment at day 0, 7, 14 and 21 days post infection. All plots were pre-gated on CD11b^−^CD11c^−^F4/80^−^B220^−^CD3^+^CD4^+^ cells. (D) Total cell number of endogenous PmpG-1-specific CD4 T cells calculated based on FACS analysis. Data shown are representative results from two independent experiments with three to five mice per time point. Bar graphs show mean number ± SEM.

Previous studies have demonstrated that pMHC class-II tetramers can be used in conjunction with tetramer enrichment, to visualize low frequency endogenous antigen-specific CD4 T cells in infected and immunized mice [22], [23]. We constructed three distinct pMHC class-II tetramers, containing I-A^b^ with a *Chlamydia*-specific epitope (RplF_51–59_, Aasf_24–32_, or PmpG-1_303–311_) bound to the MHC class-II β chain. Uninfected C57BL/6 mice contained low frequency antigen-specific CD4 T cell population specific for each *Chlamydia* epitope (Fig. 2A). However, in mice immunized subcutaneously with peptide/CFA, or infected intravenously with *C. muridarum*, an expanded population of CD44^hi^ CD4 T cells was detectable 7 days post immunization or infection (Fig. 2A). Tetramer staining was specific, as infection with *Salmonella* Typhimurium did not induce expansion of tetramer-specific CD4 T cells (Fig. 2A). Furthermore, no CD8 T cells were detected that bound to *Chlamydia* tetramers (Fig. 2A) Together, these results demonstrate that all three tetramers, RplF_51–59_:I-A^b^, Aasf_24–32_:I-A^b^, and PmpG-1_303–311_:I-A^b^ can be used to detect endogenous *C. muridarum*-CD4 T cells in vivo.

### Visualization of *Chlamydia*-specific CD4 T cell expansion to intravaginal infection

We next used the PmpG-1_303–311_:I-A^b^ tetramer to visualize clonal expansion of antigen-specific CD4 T cells during intravaginal (i.vag.) infection. We focused on PmpG-1 because CD4 T cell clonal expansion against RplF, Aasf and PmpG-1 is similar (Fig. 1B, 1C and 1D) yet PmpG-1 is a promising *Chlamydia* vaccine candidate [24]. Consistent with previous findings, *C. muridarum* bacterial loads measured by vaginal swab peaked around day 4 post-infection and steadily decreased until clearance around day 35 (Fig. 2B). To visualize the primary site of endogenous T cell priming to *Chlamydia* infection, we examined PmpG-1-specific T cell activation in multiple secondary lymphoid tissues. One week after infection, PmpG-1-specific CD4 T cells expanded in iliac lymph nodes and spleen, but were barely detectable in other non-draining lymph nodes (Fig. 2C and 2D). At day 14, endogenous PmpG-1-specific CD4 T cell expansion peaked in all secondary lymphoid tissues, and was followed by a notable contraction phase (Fig. 2D). The kinetics of the CD4 T cell response to local *Chlamydia* genital tract infection was therefore markedly delayed in comparison to systemic infection with the same pathogen (Fig. 1).

### Identification of Th1/Treg/Th17 cell subsets in *Chlamydia*-specific CD4 T cells

**Figure 3:**
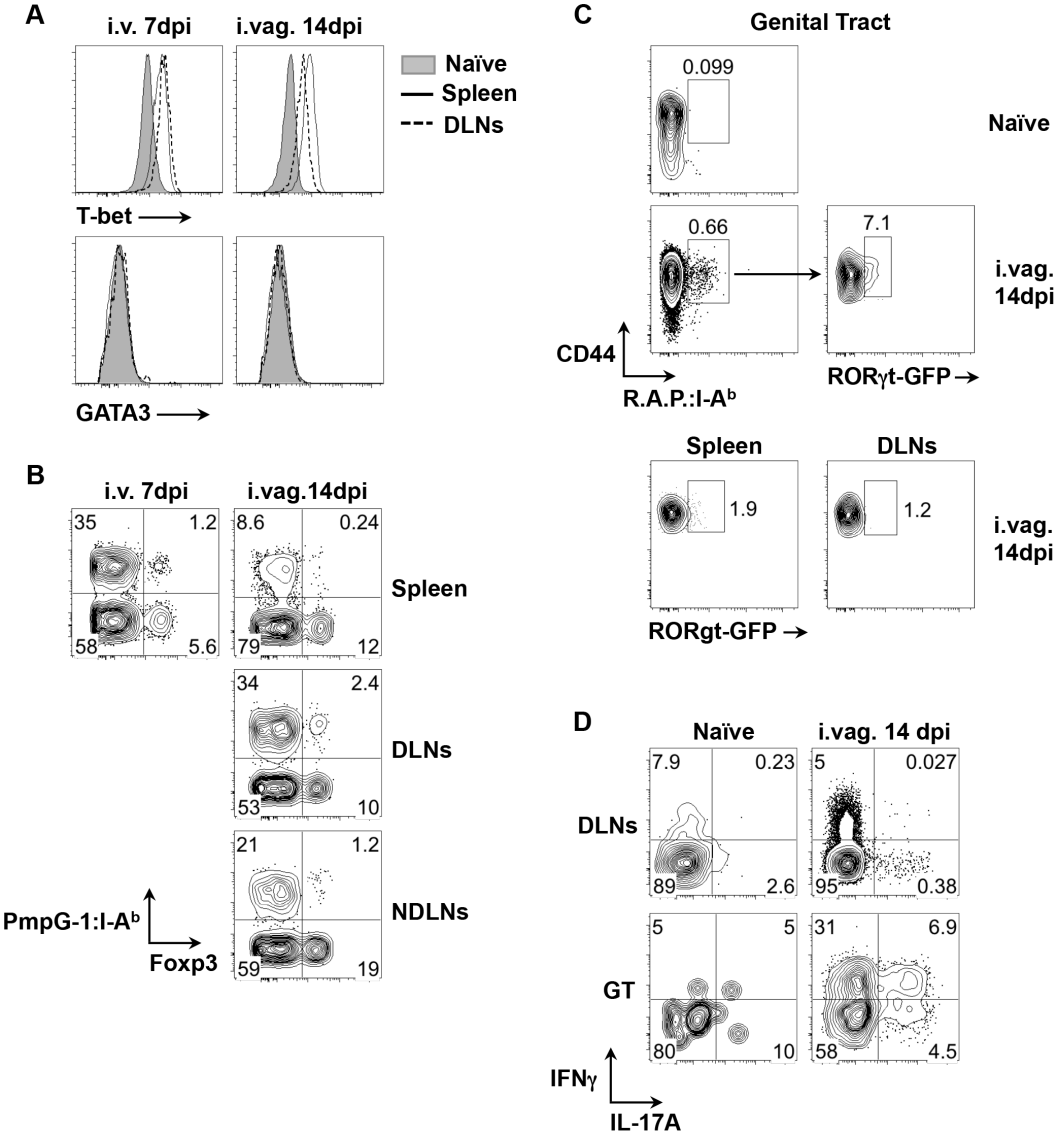
Development of Th1, Tregs and Th17 cell subsets after *C. muridarum* infections. (A) and (B) C57BL/6 mice were infected with 1×10^5^ *C. muridarum* i.v. or i.vag.. PmpG-1_303–311_:I-A^b+^ cells were enriched from spleen, DLNs and NDLNs 7 and 14 days post i.v. and i.vag. infections, respectively. (A) Histograms showing expression levels of T-bet and GATA3 on CD4^+^PmpG-1_303–311_:I-A^b+^ cells from naïve and infected mice. (B) FACS plots showing expression of Foxp3 on CD4+ cells. (C) RORγt-GFP reporter mice were infected i.vag. with 1×10^5^ *C. muridarum*. Cells from spleen, DLNs and genital tract were isolated and stained with pooled RplF_51–59_:I-A^b^, Aasf_24–32_:I-A^b^ and PmpG-1_303–311_:I-A^b^ tetramers (R.A.P.:I- A^b^). CD4^+^CD44^hi^ R.A.P.:I- A^b+^ cells were further gated for GFP expression. RORγt-GFP^−^ mice were used as a negative control for setting the GFP^+^ gates in our analysis. (D) C57BL/6 mice were infected with 1×10^5^ *C. muridarum* intravaginally. IFNγ and IL-17A production by CD4 T cells from DLNs and genital tract were detected by flow cytometry after ex vivo stimulation with phorbol 12-myristate 13-acetate and ionomycin in the presence of brefeldin A.

To examine CD4 T helper differentiation, the expression of lineage-specific transcription factors was examined in expanded CD44^hi^ PmpG-1_303–311_:I-A^b+^ CD4 T cells. Following either systemic or intravaginal infection, almost all PmpG-1-specific CD4 T cells expressed T-bet while no GATA3 expression was detected (Fig. 3A). This is consistent with previous reports that Th1 CD4 T cells are the dominant helper subset following *Chlamydia* infection [10], [11]. However, a distinct population of PmpG-1-specific CD4 T cells that co-expressed Foxp3 and T-bet was also detected after i.v. and i.vag. infection (Fig. 3B), suggesting that induced *Chlamydia*-specific Treg cells are also contained within the expanded CD4 pool. We also utilized RORγt-GFP reporter mice and combined staining with all three *Chlamydia* tetramers to examine the potential development of *Chlamydia*-specific Th17 cells after vaginal infection. While GFP-positive cells were undetectable among expanded tetramer-positive cells in the spleen or draining lymph nodes of infected mice, approximately 7% of CD4^+^CD44^hi^tetramer^+^ T cells in infected non-lymphoid tissues expressed GFP (Fig. 3C). Furthermore, stimulation of lymphocytes purified from the genital tract confirmed the presence of T cells producing both IL-17A and IFN-γ (Fig. 3D). Thus, *Chlamydia* infection of the reproductive tract induces a heterogenous T helper response that comprises expanded T-bet^+^ Th1 cells, T-bet^+^Foxp3^+^ Tregs, and Th17 cells that are enriched in infected non-lymphoid tissues.

### Reduced local CD4 expansion and *Chlamydia* dissemination in mice lacking B cells

**Figure 4:**
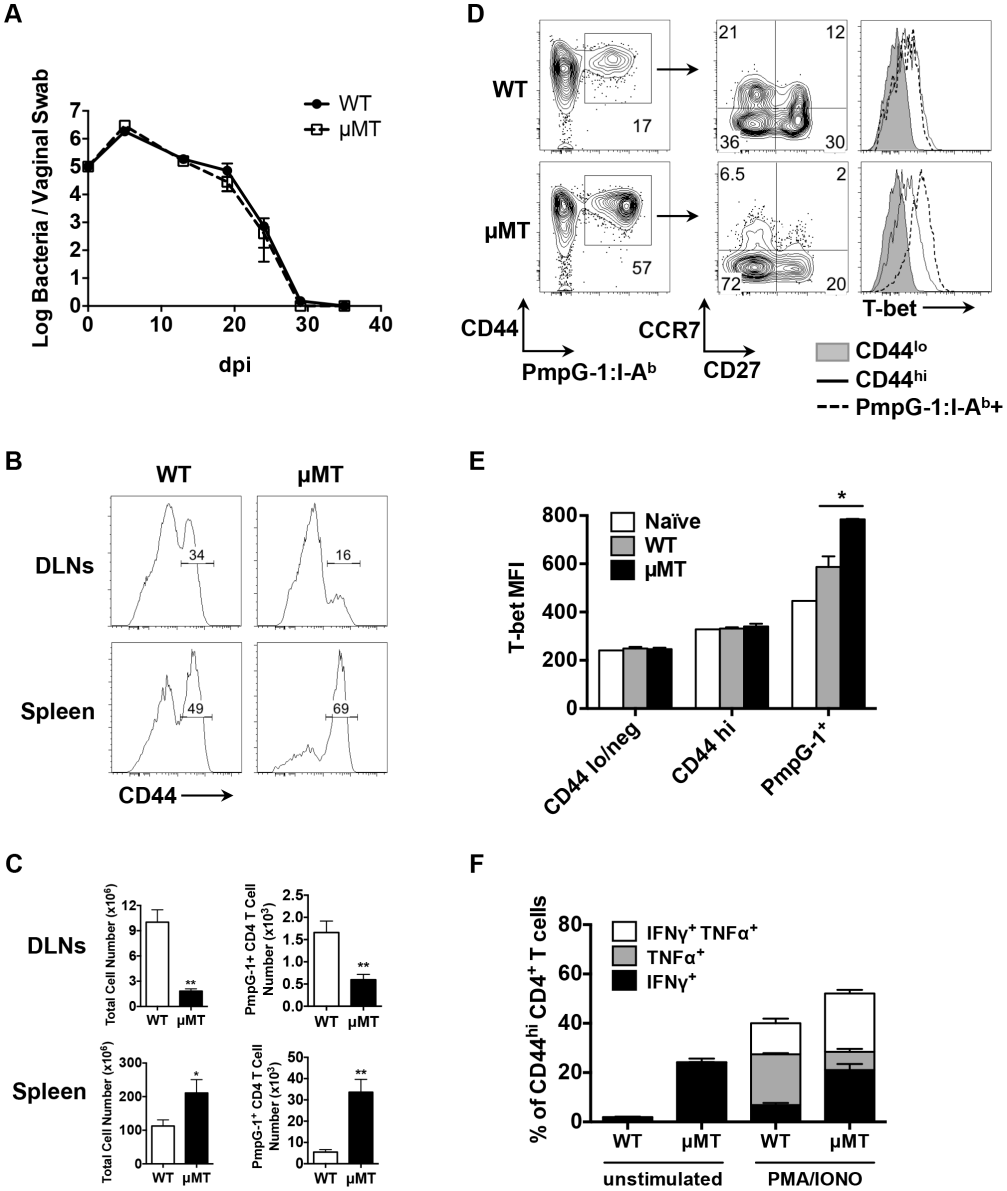
Impaired antigen-specific CD4 T cell priming in local draining lymph nodes in μMT mice after *C.muridarum* i.vag. infection. (A) Bacteria burden at lower genital tract of infected WT mice and μMT mice at various time points post infection as measured by vaginal swabs. Error bars show mean bacterial counts ± SEM. WT and μMT mice were infected intravaginally with 1×10^5^ *C. muridarum*. Fourteen days post infection, cells from spleen and DLNs were analyzed. Data shown are representative results of five independent experiments with at least three mice per group. (B) Histograms showing CD44 expression level on total CD4 T cells of infected WT and μMT mice (spleen and DLNs). (C) Total cell number and PmpG-1-specific CD4 T cell number from infected WT and μMT mice (spleen and DLNs). Error bars show mean number ± SEM. *, p<0.05; **, p<0.01. (D) and (E) Splenocytes from infected WT and μMT mice were stained and enriched for PmpG-1-specific CD4 T cells. (D) CD27 and CCR7 expression on CD4^+^CD44^hi^PmpG-1_303–311_:I-A^b+^ cells, T-bet expression on CD4^+^CD44^lo^, CD4^+^CD44^hi^ and CD4^+^CD44^hi^PmpG-1_303–311_:I-A^b+^ cells were analyzed by flow cytometry. (E) Graphs showing the mean fluorescence intensity (MFI) of T-bet expression as measured by flow cytometry in (D). Error bars show mean MFI ± SEM. *, p<0.05. (F) Splenocytes from infected WT and μMT mice were stimulated ex vivo for 4 h with PMA and ionomycin in the presence of brefeldin A. IFNγ and TNFα production by activated CD4 T cells (CD44^hi^CD4^+^) were detected by flow cytometry. The percentages of IFNγ^+^, TNFα^+^ and IFNγ^+^TNFα^+^ CD4 T cells were summarized in the graph. Error bars show mean number ± SEM.

**Figure 5:**
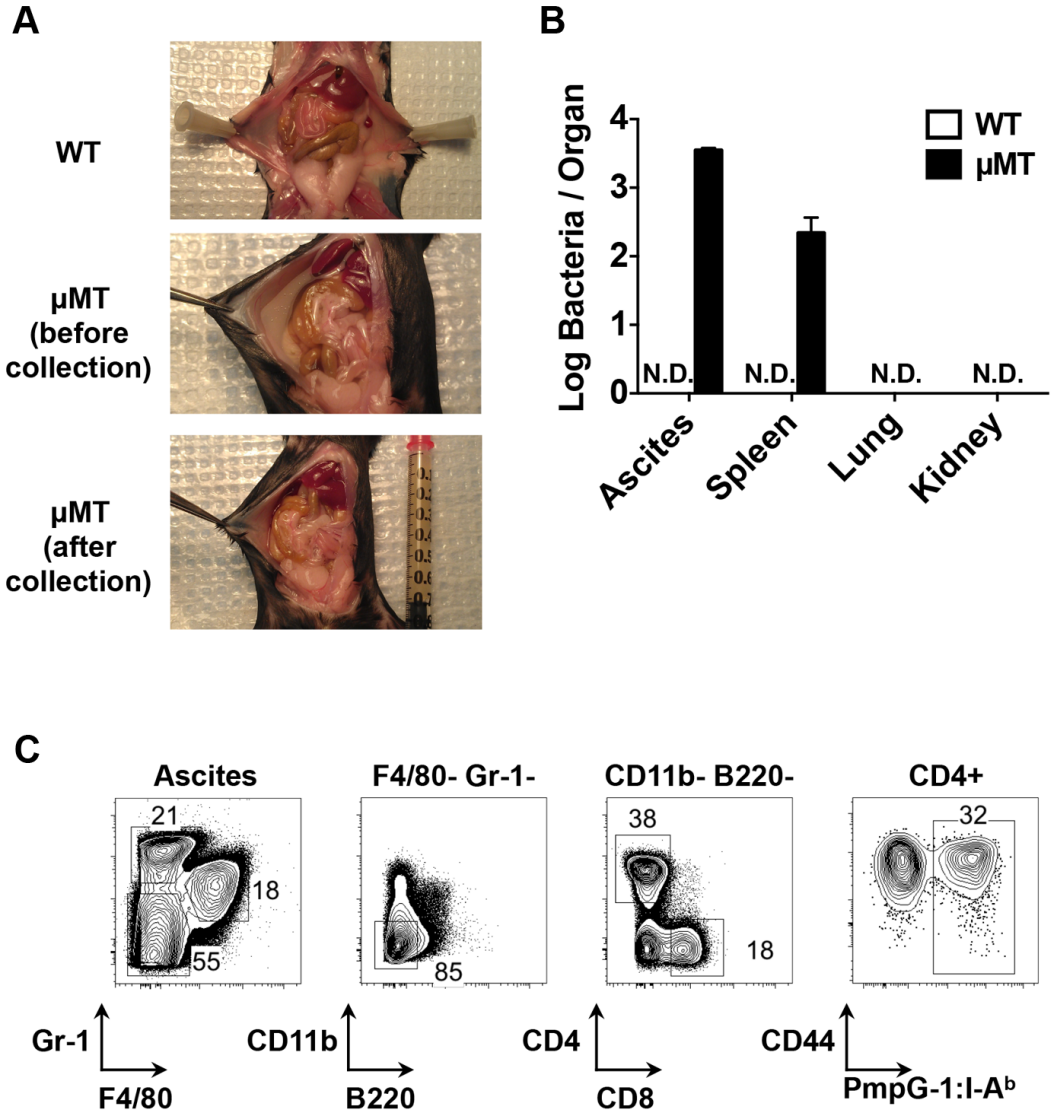
Dissemination of bacteria and development of ascites in B cell deficient mice (μMT) after *C. muridarm* i.vag. infection. C57BL/6 and μMT mice were infected with 1×10^5^ *C. muridarum* intravaginally. (A) Representative images of infected WT mice and μMT mice (before and after ascites collection). (B) Bacteria burden in the ascites (peritoneal wash of WT mice), spleen, lung and kidney from infected WT mice and μMT mice 14 days post infection. Error bars show mean bacterial counts ± SEM. (C) FACS plots showing macrophages (F4/80+), monocytes (Gr-1+), CD4 T cells (F4/80^−^Gr-1^−^CD11b^−^B220^−^CD4^+^), CD8 T cells (F4/80^−^Gr-1^−^CD11b^−^B220^−^CD8^+^) and PmpG1-specific CD4 T cells (F4/80^−^Gr-1^−^CD11b^−^B220^−^CD4^+^CD44^hi^PmpG-1_303–311_:I-A^b+^) from μMT ascites 14 days post infection. Data shown are representative results of three independent experiments with at least three mice per group.

Next, we examined bacterial shedding after vaginal infection of WT and μMT mice with *Chlamydia*. Consistent with previous reports [17], [18], bacterial shedding was unaffected by the absence of B cells (Fig. 4A). However, more detailed analysis of the local draining lymph nodes of μMT mice suggested significantly reduced activation of CD4 T cells (Fig. 4B). Indeed, using the PmpG-1_303–311_:I-A^b^ tetramer, we detected much lower clonal expansion of *Chlamydia*-specific CD4 T cells in the local draining lymph node of infected μMT mice compared to WT mice (60±12 in μMT mice vs 166±36 in WT mice, *p*<0.01; Fig. 4C). This reduced local response was also accompanied by dissemination of *Chlamydia* to the spleen and peritoneal cavity, where ascites was noted 14 days post infection (Fig. 5A and 5B). Analysis of ascites fluid from μMT mice revealed a large proportion of macrophages (F4/80^+^), monocytes (Gr-1^+^) and T lymphocytes (Fig. 5C). In addition, PmpG-1-specific CD4 T cells were abundant in ascites, demonstrating that much of the lymphocyte infiltrate into the peritoneal cavity is likely to be *Chlamydia*-specific (Fig. 5C). Thus, the absence of B cells reduces local CD4 T cell priming and allows bacterial dissemination.

### Enhanced systemic CD4 T cell priming in B cell deficient mice

In contrast to the local response, polyclonal CD4 T cells in the spleen of μMT mice displayed evidence of increased activation (Fig. 4B). Consistent with increased systemic activation, expansion of PmpG-1-specific CD4 T cells was markedly increased in the spleen of μMT mice (33600±6044 in μMT mice vs 5494±1164 in WT mice, *p*<0.001; Fig. 4C). Furthermore, *Chlamydia*-specific CD4 T cells in μMT mice expressed higher levels of T-bet and produced more IFN-γ than CD4 T cells from WT mice (Fig. 4D and E). A greater percentage of multifunctional CD4 T cells producing IFN-γ and TNF-α was also detected (Fig. 4F). Consistent with an enhanced effector response, the percentage of *Chlamydia*-specific CD4 T cells expressing CCR7 was considerably lower in μMT mice (Fig. 4D). These data suggest a compensatory systemic T cell response is induced to clear the disseminated bacteria that accompany B cell deficiency.

## Discussion

In vivo visualization studies of pathogen-specific CD4 T cell responses have typically involved adoptive transfer of monoclonal TCR transgenic T cells and have rarely focused on sexually transmitted infections [25], [26]. Here, we describe the generation of peptide-MHC class II tetramers that allow direct visualization of endogenous, polyclonal antigen-specific CD4 T cell responses to *Chlamydia* infection. Using *Chlamydia* tetramers and ELISPOT assays, we detected differences in the tempo of the initial CD4 T cell responses to systemic or vaginal challenge with *Chlamydia*. Unlike other mucosal tissues, the female genital mucosa lacks defined lymphoid structures [7], which may explain the delayed clonal expansion of CD4 T cells after *Chlamydia* genital tract infection. Alternatively, this delay may represent an unappreciated virulence mechanism of *Chlamydia* to delay early T cell priming, as has been noted during respiratory infection with MTB [27]. Thus, delayed CD4 T cell priming in the draining lymph node may be due to limited access to bacterial antigen by tissue dendritic cells or impeded migration to the local lymph nodes. Further studies will be required to examine this issue directly.

Our studies show that *Chlamydia*-specific memory CD4 T cells persist for at least one year after infection, which may suggest the presence of persistent antigen and/or persistent *Chlamydia* without culturable *Chlamydia* organisms [28], after the clearance of infectious bacteria. Interestingly, a recent study by Johnson et al suggested that the PmpG-1_303–311_ epitope can be detected on splenic APC for at least 6 months after the clearance of primary genital tract infection [29]. Our data, together with others, also support the potential role of PmpG-1 specific CD4 T cells in *Chlamydia* protective immunity [24], [29].

Our data confirm directly the previous notion that T-bet expressing Th1 cells are the predominant CD4 effector lineage among *Chlamydia*-specific CD4 T cells. However, we also identify small populations with the phenotypic marker of Tregs in the lymph node and spleen following both systemic and mucosal infection, as well as the enrichment of *Chlamydia*-specific Th17 cells at the genital mucosa itself. These data suggest that *Chlamydia*-specific Tregs are expanded to regulate the large Th1 response that develops during infection [30]. The enrichment of Th17 cells at mucosal surfaces has also been detected in other infection models [23], [31]–[33]. For example, flagellin-specific Th17 cells are predominantly enriched in the gut mucosal sites after *Salmonella* oral infection [23]. Further experimentation is required to determine whether these pathogen-specific Th17 cells contribute to *Chlamydia* clearance and/or the induction of upper genital tract pathology.

The presence of large numbers of B cells in the lower and upper genital tract during *Chlamydia* infection, as shown by both immunohistochemical staining and flow cytometry [34] (and data not shown), led us to speculate that B cells play an important role during primary *Chlamydia* infection. Indeed, our data show that in the absence of B cells, there is a marked reduction in antigen-specific CD4 T cell priming within the draining iliac lymph nodes. Given this effect of early CD4 T cell expansion it is possible that B cells participate directly in antigen presentation during the early stages of primary infection. Although this has not previously been observed during genital tract infection, a similar finding was reported after *C. muridarum* lung infection [19]. A recent study has suggested that macrophage deficiency could also account for protective defects of μMT mice against viral infections [35]. However, our observations that ascites did not occur at early time point (7 days post infection, data not shown) suggested that the phenotype we observed is mediated by altered adaptive immune mechanisms.

Although mild *Chlamydia* dissemination to other mucosal sites has been reported previously in C57BL/6 mice [36], a large number of disseminated *Chlamydia* has only previously been found in IFN-γ-deficient and SCID mice and largely attributed to deficient Th1 development [8]. B cell deficient mice therefore provide an unexpected additional model where *Chlamydia* also disseminate to non-mucosa tissues. Notably, our data provides a clear example where the marked pathology of *Chlamydia* infection does not always correlate with the inability of host to clear the infection. A likely explanation for highly efficient bacteria clearance in μMT mice is the robust systemic CD4 T cell response that may compensate for the loss of initial CD4 T cell priming within the local draining lymph nodes of the genital tract. The fact that *Chlamydia* genital tract infection can lead to ascites in the absence of B cells is also clinically relevant: *Chlamydia* infection induces ascites in patients with salpingitis and peritonitis [37]–[40], although it is unclear whether *Chlamydia* dissemination in mouse models reflects the same mechanism that leads to symptoms in human such as PID and Fitz-Hugh-Curtis syndrome. Further pathological studies are needed to understand differences of mouse and human infections.

Overall, our data demonstrate unappreciated heterogeneity of the CD4 T cell response to genital tract infection in a model where Th1 cells are essential for protective immunity. In addition, our data uncover a surprising involvement of B cells in local expansion of effector *Chlamydia*-specific T cells in the genital tract and prevention of bacterial dissemination. Greater understanding of the mechanism of *Chlamydia* dissemination from the genital tract and the T and B cell responses that restrain this spread of bacteria may reduce the risk of sequelae after *Chlamydia* genital infection in infected women.

## Materials and Methods

### Ethics statement

This study was carried out in strict accordance with the recommendations in the Guide for the Care and Use of Laboratory Animals of the National Institutes of Health. The University of California Davis is accredited by the Association for Assessment and Accreditation of Laboratory Animal Care (AAALAC). All animal experiments were approved by University of California Davis Institutional Animal Care and Use Committee (IACUC) (Protocol number 16612).

### Mice

C57BL/6 (B6) mice were purchased from the National Cancer Institute (Frederick, MD) and The Jackson Laboratory (Bar Harbor, ME). μMT mice were purchased from The Jackson Laboratory (Bar Harbor, ME). RORγt-GFP reporter mice were obtained from Dr. Marc Jenkins (University of Minnesota). Mice used for experiments were 6–12 weeks old, unless otherwise noted. All mice were maintained in accordance with University of California Davis Research Animal Resource guidelines.

### Bacteria

*Chlamydia muridarum* strain Nigg II was purchased from ATCC (Manassas, VA). The organism was cultured in HeLa 229 cells in Dulbecco's Modified Eagle Medium (DMEM) (Life Technologies, Grand Island, NY) supplemented with 10% fetal bovine serum (FBS). Elementary bodies (EBs) were purified by discontinuous density gradient centrifugation as previously described and stored at −80 degree [41]. The number of IFUs of purified EBs was determined by infection of HeLa 229 cells and enumeration of inclusions that were stained with anti-*Chlamydia* MOMP antibody. Heat-killed EBs (HKEBs) were prepared by heating at 56°C for 30 min. A fresh aliquot was thawed and used for every infection experiment. *Salmonella enterica* serovar *Typhimurium* strains BRD509 (AroA^−^D^−^) were kindly provided by Dr. D. Xu (University of Glasgow, Glasgow, U.K).

### *Chlamydia* infection and enumeration

For systemic infection, mice were injected intravenously in the lateral tail vein with 1×10^5^ *C. muridarum*. To enumerate the bacteria burden in tissues, the spleen, liver and kidney, were crushed in 5 mL SPG buffer and tissue homogenate was placed in a tube with glass beads to disrupt cells. After shaking for 5 min, and centrifugation at 500 g for 10 minutes, supernatants were collected and serial dilutions were plated on HeLa 229 cells. For intravaginal infections, estrus was synchronized by subcutaneous injection of 2.5 mg medroxyprogesterone acetate (Greenstone, NJ) 7 days before infection. 1×10^5^ C. muridarum in 5 µL SPG buffer were then deposited into vaginal vaults. To enumerate bacteria, vaginal swabs were collected, shaken with glass beads, and serial dilutions were plated on HeLa 229 cells.

### ELISPOT assay

Spleen and lymph nodes (axillary, brachial, inguinal and mesenteric) were harvested and a single-cell suspension prepared. After RBC lysis, CD4^+^ T cells were enriched using LS MACS columns and anti-CD4 magnetic beads (Miltenyi Biotec, Auburn, CA). Enriched CD4^+^ T cells were incubated with irradiated APCs from naive mice in the presence of 10 µM *Chlamydia* peptide (RplF_51–59_, FabG_157–165_, Aasf_24–32_ or PmpG-1_303–311_) in 96-well ELISPOT plates (Millipore, Billerica, MA) that had been pre-coated with purified anti-IFN-γ (BD Biosciences, San Diego, CA). The RplF_51–59_, FabG_157–165_, Aasf_24–32_ and PmpG-1_303–311_ epitopes used for stimulation have been described previously [21], [42]. After 20 h of incubation at 37°C, cells were washed and cytokine spots developed using biotinylated anti-IFN-γ (BD Biosciences), AKP Streptavidin (BD Biosciences), and 1-Step NBT/BCIP substrate (Thermo Scientific, Waltham, MA). Cytokine spots were counted using an ImmunoSpot S5 Core Analyzer (C.T.L., Shaker Heights, OH), and the total number of IFN-γ-producing CD4+ T cells per spleen was calculated.

### Construction of pMHCII tetramers

The methodology for construction of pMHCII tetramers has been described in detail [22]. Briefly, biotinylated I-A^b^ monomers containing a covalently linked *C. muridarum* peptide (RplF_51–59_, Aasf_24–32_ or PmpG-1_303–311_) were expressed by S2 Drosophila insect cell lines and cultured in a Wave Bioreactor (GE Healthcare Biosciences, Pittsburgh, PA). After purification, I-A^b^ monomers were tetramerized by co-incubated with fluorochrome-conjugated streptavidin at a pre-determined optimal ratio at room temperature for 30 min [22]. To test for tetramer specificity, C57BL/6 mice were immunized with each of the three peptides and CFA (Sigma-Aldrich, St. Louis, MO). Seven days post-immunization, draining lymph nodes were isolated and tetramer positive cells enriched using the methodology described below.

### Tetramer enrichment and flow cytometry

Spleen and LNs were harvested from naïve or infected mice. Single cell suspensions were prepared in FACS buffer (PBS with 2% FCS) and stained with tetramers in Fc block (culture supernatant from the 24G2 hybridoma, 2% mouse serum, 2% rat serum, and 0.01% sodium azide) for 1 h at room temperature in the dark. Cells were then washed and tetramer positive cells enriched via LS MACS columns and anti-fluorochrome magnetic beads (Miltenyi Biotec, Auburn, CA). Bound and unbound fractions were stained with a panel of monoclonal antibodies (listed below) and analyzed on a FACSCanto or an LSRFortessa flow cytometer (BD Biosciences, San Jose, CA). To stain for intracellular transcription factors and cytokines, cells were left untreated or stimulated with phorbol 12-myristate 13-acetate (PMA, 50 ng/ml), ionomycin (200 ng/ml) in the presence of brefeldin A (10 µg/ml) for 4 h at 37°C. After surface staining, cells were fixed, permeablized and stained using the Foxp3 staining Kit (eBioscience, San Diego, CA). Antibodies for staining included FITC-CD11b, CD11c, F4/80, B220, TNFα; PerCP-eFlour710-CD4; APC-CCR7; eFlour660-T-bet; Alexa700-CD44; eFlour450-CD3, Foxp3, IFN-γ (eBioscience, San Diego, CA); FITC-IL-17A and APC-Cy7-CD8 (BD Biosciences, San Diego, CA). Flow data were analyzed using FlowJo software (Tree Star, Ashland, OR) and endogenous, tetramer-specific CD4 T cells were identified using a previously published gating strategy [22].

### Statistical analysis

All data sets were analyzed by unpaired Student's *t*-test using Prism (GraphPad Software, La Jolla, CA). A p value<0.05 were considered statistically significant.
